# *In vitro* and *in vivo* effects of *Pelargonium sidoides DC.* root extract EPs^®^ 7630 and selected constituents against SARS-CoV-2 B.1, Delta AY.4/AY.117 and Omicron BA.2

**DOI:** 10.3389/fphar.2023.1214351

**Published:** 2023-07-26

**Authors:** Jackson Emanuel, Jan Papies, Celine Galander, Julia M. Adler, Nicolas Heinemann, Kathrin Eschke, Sophie Merz, Hannah Pischon, Ruben Rose, Andi Krumbholz, Žarko Kulić, Martin D. Lehner, Jakob Trimpert, Marcel A. Müller

**Affiliations:** ^1^ Institute of Virology, Charité—Universitätsmedizin Berlin, Corporate Member of Freie Universität Berlin, Humboldt-Universität zu Berlin, Berlin, Germany; ^2^ German Center for Infection Research (DZIF), Partner Site Charité, Berlin, Germany; ^3^ Institut für Virologie, Freie Universität Berlin, Berlin, Germany; ^4^ IDEXX Laboratories, Kornwestheim, Germany; ^5^ Institute for Infection Medicine, Kiel University and University Hospital Schleswig-Holstein, Kiel, Germany; ^6^ Labor Dr. Krause und Kollegen MVZ GmbH, Kiel, Germany; ^7^ Preclinical R&D, Dr. Willmar Schwabe GmbH and Co. KG, Karlsruhe, Germany

**Keywords:** SARS-CoV-2, coronavirus, *Pelargonium sidoides*, EPs 7630, drug repurposing, immune modulation, COVID-19, cytokine storm

## Abstract

The occurrence of immune-evasive SARS-CoV-2 strains emphasizes the importance to search for broad-acting antiviral compounds. Our previous *in vitro* study showed that *Pelargonium sidoides DC.* root extract EPs^®^ 7630 has combined antiviral and immunomodulatory properties in SARS-CoV-2-infected human lung cells. Here we assessed *in vivo* effects of EPs^®^ 7630 in SARS-CoV-2-infected hamsters, and investigated properties of EPs^®^ 7630 and its functionally relevant constituents in context of phenotypically distinct SARS-CoV-2 variants. We show that EPs^®^ 7630 reduced viral load early in the course of infection and displayed significant immunomodulatory properties positively modulating disease progression in hamsters. In addition, we find that EPs^®^ 7630 differentially inhibits SARS-CoV-2 variants in nasal and bronchial human airway epithelial cells. Antiviral effects were more pronounced against Omicron BA.2 compared to B.1 and Delta, the latter two preferring TMPRSS2-mediated fusion with the plasma membrane for cell entry instead of receptor-mediated low pH-dependent endocytosis. By using SARS-CoV-2 Spike VSV-based pseudo particles (VSVpp), we confirm higher EPs^®^ 7630 activity against Omicron Spike-VSVpp, which seems independent of the serine protease TMPRSS2, suggesting that EPs^®^ 7630 targets endosomal entry. We identify at least two molecular constituents of EPs^®^ 7630, i.e., (−)-epigallocatechin and taxifolin with antiviral effects on SARS-CoV-2 replication and cell entry. In summary, our study shows that EPs^®^ 7630 ameliorates disease outcome in SARS-CoV-2-infected hamsters and has enhanced activity against Omicron, apparently by limiting late endosomal SARS-CoV-2 entry.

## 1 Introduction

Since its introduction into humans in late 2019 and global spread throughout 2020, SARS-CoV-2 has become endemic in the human population and remains an important public health challenge. Successively emergent SARS-CoV-2 variants, including Delta (e.g., AY.4, AY.117) and Omicron (e.g., BA.1, BA.2, BA2.75, XBB.1.5), have properties that confer resistance to existing antiviral therapies. Specifically, vaccines and monoclonal antibody therapies, which previously elicited strong neutralization of SARS-CoV-2, show significantly reduced neutralization of currently circulating variants (Davis et al., 2021; Edara et al., 2022; Tada et al., 2022; Wang et al., 2023). Remaining methods for controlling SARS-CoV-2/COVID-19 show varying degrees of efficacy (Mohamed et al., 2022), and may be affected by the emergence of future SARS-CoV-2 variants. This emphasizes the continuing need for new or re-purposed, broad-acting antiviral therapeutics that can overcome viral resistance.

COVID-19 can result in severe inflammatory responses and immunological dysregulations. The release of pro-inflammatory cytokines and infiltration of immune cells into the lungs are key characteristics of severe COVID-19-associated acute respiratory distress syndrome (Pelaia et al., 2020; Hu et al., 2021; Hönzke et al., 2022). Several COVID-19-related immune markers were proposed as potential druggable targets in the treatment of COVID-19, including members of the CXC chemokine family that promote immune cell chemotaxis to the site of infection (Didangelos, 2020; Gudowska-Sawczuk and Mroczko, 2022). Therapeutics which reduce the host pro-inflammatory response by limiting the release of cytokines, such as IL-8, CXCL9, and IP-10 (Zhao et al., 2020; Callahan et al., 2021; Matsushima et al., 2022) may therefore reduce excessive immune cell infiltration of the lungs and improve host respiratory function. Anti-SARS-CoV-2 therapeutics should ideally combine antiviral and immunomodulatory properties, so that both the virus and the symptoms/disease can be effectively targeted with a single treatment.

*Pelargonium sidoides DC.* is a medicinal plant indigenous to South Africa (Brendler and van Wyk, 2008). In a previous study, we characterized the *P. sidoides DC.* root extract EPs^**®**^ 7630, and found that it has both antiviral and anti-inflammatory effects on SARS-CoV-2 infection *in vitro* (Papies et al., 2021), making it a suitable candidate for further preclinical *in vivo* model-based investigations. The Syrian hamster is a widely accepted model that allows to fully evaluate COVID-19 and its effects on disease pathology connected to a dysregulated immune response (Osterrieder et al., 2020). However, detailed investigations of immunomodulatory effects in hamster models are challenging due to a lack of validated immunological tools. Sophisticated human respiratory *in vitro* models might serve as a complementary model to investigate putative antiviral and anti-inflammatory effects of immunomodulatory compounds during SARS-CoV-2 infections at the actual site of replication.

EPs^**®**^ 7630, a proprietary hydroethanolic extract of *P. sidoides DC.* (Geraniaceae) roots, is the active principle in herbal medicinal products used for the treatment of respiratory tract infections such as acute bronchitis or common cold (Matthys et al., 2003; Chuchalin et al., 2005; Kamin et al., 2010a; Kamin et al., 2010b; Riley et al., 2019). It is composed of carbohydrates, minerals, peptides, purine derivatives, highly substituted benzopyranones, and oligo- and polymeric prodelphinidins (Schötz et al., 2008). Subfractionation of multicomponent entities for pharmacological testing can help to distinguish antiviral and immunomodulatory effects of plant extracts and can be carried out following different strategies. EPs^**®**^ 7630 subfractions tested by Papies *et al.* were generated by ultrafiltration, generating fractions containing molecules of different molecular sizes. In contrast, an orthogonal strategy for subfractionation involves selecting single characteristic molecules of EPs^**®**^ 7630 representing different natural product classes. For example, umckalin and umckalin sulfate are characteristic representatives of benzopyranones (coumarins) found in EPs^**®**^ 7630. Taxifolin sulfate and other flavonoid sulfates were recently discovered as genuine constituents in *P. sidoides* root extract EPs^**®**^ 7630 (Kulić et al., 2022). Taxifolin has been identified *in silico* as a potential inhibitor of SARS-CoV-2 protease (Fischer et al., 2020) and RNA-dependent RNA polymerase (Kandeel et al., 2020). In addition, catechins such as gallocatechin, epigallocatechin and epigallocatechin gallate were identified in EPs^**®**^ 7630. Epigallocatechins from green tea, for example, were previously shown to inhibit SARS-CoV-2 and other CoV entry (Henss et al., 2021; Liu et al., 2021; Ohishi et al., 2022). Furthermore, EPs^**®**^ 7630 contains prodelphinidin B1 (Epigallocatechin-4β→8-gallocatechin) and prodelphinidin B4 (Gallocatechin-4α→8-epigallocatechin) which are dimeric prodelphinidins covering different stereochemical configurations of this substance class. Although multiple subfractions of EPs^**®**^ 7630 were found to contribute to its activity (Papies et al., 2021), the relative contribution of individual molecular components of EPs^**®**^ 7630 has not yet been determined. Since SARS-CoV-2 variant Omicron has been shown to prefer an altered cellular entry mechanism compared to preceding variants (Meng et al., 2022; Willett et al., 2022), it may be the case that entry-targeting compounds exhibit differential activity against SARS-CoV-2 variants. Initial reports suggest that Omicron exhibits less TMPRSS2-dependent plasma membrane fusion and favors TMPRSS2-independent late endosomal entry (Meng et al., 2022; Willett et al., 2022). Thus, examining the degree to which EPs^**®**^ 7630 and its components inhibit the entry of phenotypically different SARS-CoV-2 variants provides a method of studying the antiviral mechanism of EPs^**®**^ 7630 in greater detail.

## 2 Materials and methods

### 2.1 *Pelargonium sidoides DC.* Extract EPs^®^ 7630 and individual constituents

For all experiments, a sample of a production batch (EXCh. 878) of EPs^**®**^ 7630, a dried extract of *P. sidoides DC.* roots (1:8–10), extraction solvent: ethanol 11% (w/w) was used. 80% of the roots used for the aforementioned production batch were collected from wild plant populations and 20% were harvested from plantations in South Africa. Prior to extraction, the dried plant material was tested in an array of DNA-based and phytochemical methods to confirm the quality and identity of the herbal material. Pharmacognosy was done by the quality control department of Dr. Willmar Schwabe GmbH and Co. KG. Voucher specimens of every lot are deposited in the Department of Pharmacognosy to be retained for 10 years. Chemical fingerprinting of the used EPs^®^ 7630 batch according to the Consensus statement on the Phytochemical Characterization of Medicinal Plant extracts (Heinrich et al., 2022) by three different methods (NMR, HPLC, GPC) was already carried out in our previous study (Papies et al., 2021).

Umckalin (7-Hydroxy-5,6-dimethoxy-2H-1-benzopyran-2-one, cpd A) (Table 1) and umckalin sulfate (5,6-Dimethoxy-7-(sulfooxy)-2H-1-benzopyran-2-one, cpd B) were isolated as described previously (Schötz et al., 2008). (−)-Epigallocatechin (cpd C) was purchased from Interchim S.A., France. Prodelphinidin B1 (Epigallocatechin-4β→8-gallocatechin, cpd D) and prodelphinidin B4 (Gallocatechin-4α→8-epigallocatechin, cpd E) were isolated by fractionation of the low molecular weight prodelphinidin fraction containing dimers and trimers, as previously described (Schötz and Nöldner, 2007). The single compounds were purified by dissolving the aforementioned dimer/trimer fraction in methanol to a concentration of 12% (w/v) and loading the solution on a Toyopearl HW-40S column (length = 45 cm, diameter = 2.5 cm) preconditioned with methanol, which was previously saturated with N_2_ gas. Fractions were eluted from the resin by an isocratic N_2_-saturated methanol flow. Fractions were checked by thin layer chromatography and pooled to yield the single compounds. (−)-Epigallocatechin gallate (cpd F) was purchased from TCI Deutschland GmbH, Germany. (+)-Taxifolin (cpd G) was purchased from Merck KGaA, Darmstadt, Germany. The purity of the purchased compounds were taken from the vendors’ specifications and had an >98% HPLC purity. The purity of the isolated substances was checked by ^1^H-NMR spectroscopy (Supplementary Figure S1), using the same instrument as described previously (Papies et al., 2021). Apart from residual solvents (water, ethanol), no additional impurities could be detected for umckalin and umckalin sulfate in the NMR spectra. For prodelphinidin B1 and B4, some minor impurities from oligo-/polymeric prodelphinidins were detectable as signal bulges below the sharp signals of the respective pure dimer, in addition to residual solvent (water). Thus, the purity of all substances can be considered suitable for the study.

**TABLE 1:** EPs^®^ 7630 small molecules.

Compound (cpd)	Name	Content in EPs^®^ 7630 (m/m)		Substance class
cpd A	umckalin	∼0.2–0.8%	in batch 878^a^	benzopyranone
cpd B	umckalin sulfate	∼0.2–0.8%	Σ = 0.75%	benzopyranone
cpd C	(−)-epigallocatechin	<0.3%		catechin
cpd D	prodelphinidin B1	<0.7%		prodelphinidin
cpd E	prodelphinidin B4	<0.7%		prodelphinidin
cpd F	(−)-epigallocatechin gallate	not analyzed^b^		catechin
cpd G	(+)-taxifolin	∼0.1% (Kulić et al., 2022)		flavonoid

^a^ As specified in the certificate of analysis for the batch. The contents of the catechins and prodelphinidins were calculated from isolation yields (data not shown).

^b^ (−)-epigallocatechin gallate has been described for a 50% methanolic *Pelargonium sidoides* extract (Savickiene et al., 2018). Quantification of gallic acid after hydrolysis of ca. 0.01% in EPs**®** 7630 may correspond to a possible content of ca. < 0.027% epigallocatechin gallate.

Test solutions were prepared as follows: EPs^®^ 7630 was suspended in DMEM for a stock concentration of 2 mg/mL, and serially diluted in assay medium to achieve the required working concentration. All compounds listed in Table 1 were prepared by suspension in DMSO for stock concentrations of 10 mg/mL, and serially diluted in assay medium to achieve the required working concentration. DMSO vehicle controls contained an equivalent amount of DMSO to the amount of DMSO in wells treated with the highest concentration of compound in each assay.

### 2.2 Ethics statement

*In vivo* experiments were performed in the biosafety level three (BSL-3) facility at the Institut für Virologie, Freie Universität Berlin, Germany. Animal work was approved and executed in compliance with all applicable institutional, national and international regulations (Landesamt für Gesundheit und Soziales Berlin, permit number 0086/20).

### 2.3 Animal husbandry

Syrian hamsters (*Mesocricetus auratus;* breed RjHan:AURA) were purchased from Janvier Labs at 10 weeks of age. The animals were kept in individually ventilated cages (IVCs) in groups of 1–3 hamsters and had 1 week to get used to the housing conditions. Food and water were offered *ad libitum*. During the experiment, the cage temperature was constantly between 22°C and 24°C with a relative humidity between 40% and 55%.

### 2.4 Infection experiments

Syrian hamsters were randomly assigned into groups of 9 animals (40%–60% female hamsters per group). Intranasal infection with 10^5^ plaque forming units (PFU) of SARS-CoV-2 (BetaCoV/Munich/BavPat1/2020) in 60 µL minimal essential medium (MEM) was performed under general anesthesia. EPs^**®**^ 7630 was applied in strawberry syrup orally at a dose of 50 mg/kg body weight twice daily. One treatment group received the first dosage of EPs^**®**^ 7630 1 day before infection, while the second therapeutic and vehicle treatment group were started on the day of infection. The vehicle group received strawberry syrup without EPs^**®**^ 7630. The treatment group that started on the day of infection additionally received EPs^®^ 7630 intranasally at 5 mg/mL together with the virus inoculum (60 µL total volume).

The rationale to include an additional intranasal administration of EPs^®^ 7630 in one of the treatment groups was based on results from our previous study (Papies et al., 2021). In that study fractionation of EPs^®^ 7630 demonstrated highest antiviral activity in fractions containing oligomeric proanthocyanidins with expected low oral bioavailability. We assumed that local administration at the site of infection to bypass low systemic bioavailability could increase antiviral activity. We decided to include a single intranasal administration concomitantly with the virus inoculum as a first proof-of-principle approach to assess whether topical mucosal administration holds any promise as a future development option.

Infected hamsters were checked twice daily for development of clinical symptoms and body weight loss. Euthanasia was scheduled on day 2, 4 and 7 after infection. Animals were anesthetized with medetomidine (0.15 mg/kg body weight), midazolam (2 mg/kg body weight), and butorphanol (2.5 mg/kg body weight) prior to euthanasia. Lungs, serum, EDTA blood and oropharyngeal swabs were collected to conduct virological and histopathological analysis.

### 2.5 RNA extraction and qPCR

RNA was extracted from oropharyngeal swabs and 25 mg homogenized lung tissue using innuPREP Virus DNA/RNA Kit (Analytic Jena, Jena, Germany) according to the manufacturer’s instructions. NEB Luna universal Probe One-Step RT-qPCR Kit (New England Biolabs, Ipswich, MA, United States) was used to perform qPCR with cycling conditions of 10 min at 55°C for reverse transcription, 3 min at 94°C for activation of the enzyme, and 40 cycles of 15 s at 94°C and 30 s at 58°C on a qTower G3 cycler (Analytic Jena, Jena, Germany) in sealed qPCR 96-well plates. To monitor virus growth, SARS-CoV-2 RNA was quantified in cell culture supernatants by RT-qPCR targeting the SARS-CoV-2 E gene, as described previously (Corman et al., 2020).

### 2.6 Plaque assay for *in vivo* experiments

To quantify replication-competent infectious virus, titrations were performed from 50 mg lung tissue and oropharyngeal swabs. For sample preparation, swabs were thawed, kept in virus transport medium (PBS with 25 mg/L enrofloxacin and 10 mg/L voriconazole) for 30 min and vortexed 3 times during incubation. The organ samples were homogenized in a bead mill procedure with ceramic beads (Analytic Jena). Thereafter, 10-fold serial dilutions were prepared starting from −1 to −6 and plated on VeroE6 cells grown in 12-well plates. The plates were incubated for 2 h at 37°C and subsequently overlaid with MEM medium containing 1.5% carboxymethylcellulose sodium (Sigma Aldrich, St. Louis, MO, United States). The plates were fixed with 4% PBS-buffered formaldehyde solution 72 h after infection. 0.75% methylene blue was used to visualize and manually count plaques. The assay-specific limit of detection is 10 PFU/50 mg tissue. All titration experiments were performed in duplicate wells. For samples without detectable plaques, a value of 5 PFU, corresponding to half the assay limit of detection was assigned to allow log-transformation of data.

### 2.7 Histopathology

The left lung lobe was prepared for histopathological examination as previously described (Osterrieder et al., 2020). After careful preparation, it was fixed in PBS-buffered 4% formaldehyde solution for 48 h, embedded in paraffin and cut at 2 μm thickness. Subsequently, the slides were stained with hematoxylin and eosin (H&E) as previously published (Bertzbach et al., 2021).

### 2.8 Cell lines

Calu-3 (ATCC HTB-55), VeroFM (ATCC CCL-81), A549-ACE2, A549-ACE2-TMPRSS2 (Widera et al., 2021) were grown in Dulbecco’s Modified Eagle’s Medium (DMEM) supplemented with 10% fetal bovine serum (FBS), 1% non-essential amino acids, and 1% sodium pyruvate at 37°C and 5% CO_2_. VeroE6 (ATCC CRL-1586) and VeroE6-TMPRSS2 (NIBSC 100978) cells were cultured in minimal essential medium (MEM) containing 10% fetal bovine serum, 100 IU/mL penicillin G, and 100 μg/mL streptomycin. For VeroE6-TMPRSS2 cell culture (NIBSC 100978), the medium also contained 1,000 μg/mL geneticin (G418) to select for cells expressing TMPRSS2. The cells were incubated at 37°C and 5% CO_2_. All cell lines were cultivated under sterile laboratory conditions and tested for simian virus 5 and *mycoplasma* contamination as described previously (Biesold et al., 2011).

### 2.9 Virus strains and infection

The SARS-CoV-2 strain Munich/2020/984 was isolated from a respiratory swab obtained from the early 2020 Munich patient cohort (GenBank: MT270101; GISAID: EPI_ISL_406862). The Delta AY.4 variant was isolated from a patient in Cotonou, Benin in July 2021 (GISAID: EPI_ISL_4566935) (Yadouleton et al., 2022). The Omicron BA.2 variant was isolated from a patient in Schleswig-Holstein, Germany in January 2022 (GISAID: EPI_ISL_9553926).

Stocks for animal experimentation were generated on VeroE6-TMPRSS2 cells and titrated on VeroE6 cells. Prior to animal infection, all virus stocks were stored at −80°C.

Stocks for *in vitro* experimentation were both generated and titrated on VeroE6 cells. For SARS-CoV-2 infection of cell cultures, between 2 × 10^5^ and 3 × 10^5^ cells per mL were seeded in 6-well plates or 24-well plates. After 24 h, cells were infected with SARS-CoV-2 in a serum-free medium. After 1 h, virus dilutions were removed, and the wells were washed twice with PBS and refilled with DMEM (supplemented as described previously). Samples were taken at the indicated time points. The full sequence identity of B.1, Delta AY.4, and Omicron BA.2 SARS-CoV-2 stocks for *in vitro* experiments was confirmed with NGS and RT-PCR/Sanger sequencing, and can be made available upon request. Lineage assignment was verified with the Pangolin Web Application (v4.2, pangolin-data version v1.18.1.1) (O’Toole et al., 2021).

All virus infection experiments were conducted under biosafety level 3 conditions with enhanced respiratory personal protection equipment.

### 2.10 Plaque assay for *in vitro* experiments

SARS-CoV-2 plaque-forming units (PFU) were quantified by plaque titration on VeroE6 cells as described before (Dulbecco, 1952; Herzog et al., 2008; Forcic et al., 2010). Briefly, monolayers of VeroE6 cells were seeded in 24-well plates with ∼90% confluency and washed with PBS, incubated with serial dilutions of SARS-CoV-2-containing cell culture supernatants, and overlaid with 1.2% Avicel in DMEM 24 h after seeding. 72 h post-infection, cells were fixed with 6% formalin and visualized by crystal violet staining. The assay-specific limit of detection is 50 PFU/mL. All titration experiments were performed in duplicate wells.

### 2.11 Primary respiratory epithelial cell infection assay

Human nasal and bronchial airway epithelial cells (AEC) MucilAir™ cell cultures containing club, ciliated, and basal cells were purchased from Epithelix Sàrl (Geneva, Switzerland). Both cell cultures were derived from pooled patient material from healthy donors. Cells were cultivated in 24-well plates under air-liquid-interface conditions using transwell^®^ inserts and predefined serum-free MucilAir™ culture medium obtained from Epithelix at 37°C and 5% CO_2_. Mucus was removed by gentle washing of the apical surface with PBS multiple times 2 days before performing experiments to ensure uniform conditions of the mucous surface in each well.

For infection of MucilAir™ cultures, cells were inoculated on the apical side with SARS-CoV-2 B.1, SARS-CoV-2 AY.4, or SARS-CoV-2 BA.2 diluted in MucilAir™ medium using an MOI of 0.005 with and without EPs^®^ 7630 (100 μg/mL) treatment at 37°C for 2 h. After inoculation, the virus-containing solution was removed and the apical side was washed gently with PBS three times to remove non-attached virus. For sample taking, the apical side of the AEC was incubated with 250 µL MucilAir™ medium for 20 min, which was subsequently removed and frozen at −80°C until analysis. Supernatants were analyzed by plaque assay between 0 and 72 h post-infection.

### 2.12 Cytokine quantification

To assess cytokine levels in primary bronchial AEC (bAEC) supernatant, 25 µL of supernatant were sampled before infection and at 24 h post-infection with SARS-CoV-2. Cytokines were quantified using a Human Cytokine/Chemokine/Growth Factor Panel A 48-Plex Premixed Magnetic Bead Multiplex Assay (Merck Millipore, Burlington, MA, United States), using the Luminex MAGPIX System in 96-well plate format, according to the manufacturer’s instructions. Plate washing steps were performed using the HydroFlex Microplate Washer (Tecan, Männedorf, Switzerland). Calibration and verification checks (Bio-Techne, Minneapolis, MN, United States) were met for all of the analytes.

### 2.13 VSV-pseudo-particle assay

SARS-CoV-2 spike (S) protein-dependent viral entry was assessed using an established vesicular stomatitis virus (VSV) pseudo-particle (VSVpp) assay as described elsewhere (Kleine-Weber et al., 2019; Hoffmann et al., 2020; Zettl et al., 2020). Briefly, A549-ACE2 cells, A549-ACE2-TMPRSS2 and Calu-3 cells were seeded in DMEM in 96-well plates with a density of 50%–70% 24 h before infection with VSVpp. For pre-treatment, medium was removed 2 h before infection and replaced by fresh DMEM containing EPs^®^ 7630, compounds listed in Table 1, camostat mesylate, niclosamide, or DMSO as vehicle control for niclosamide at the indicated concentrations. After pre-incubation, medium was removed and fresh DMEM containing VSVpp carrying the SARS-CoV-2 S. S variants included B.1 (encoding the S protein derived from BetaCoV/Munich/BavPat1/2020; GISAID: EPI_ISL_406,862), Delta AY.117 (encoding a protein identical to GenBank: QYN98425.1), Omicron BA.2 (encoding a protein identical to GenBank: UHU97100.1), Alpha B.1.1.7 (encoding the S protein derived from BetaCoV/Baden-Wuerttemberg/ChVir21528/2020; EPI_ISL_754174), and Beta B.1.351 (encoding the S protein derived from Baden-Wuertemberg/ChVir22131/2021; EPI_ISL_862149). All wells were inoculated with the equivalent titer of respective VSVpp, which was previously found to be in the dynamic range of the luciferase assay. 19 amino acids on the C-terminus of the S protein were omitted from all variants to obtain optimal VSVpp incorporation and expression. Plates were centrifuged for 30 min at 4°C and 300 *g* to achieve synchronized infection. After additional incubation for 90 min at 37°C, 5% CO_2_, compound-containing medium was added to the cells. To measure luciferase production, which correlates with successful viral entry, cell lysates were prepared after 24 h using passive lysis buffer (Promega, Madison, WI, United States). Lysates were then transferred to opaque 96-well plates and luminescence was measured in a BioTek multi-well plate reader using Luciferase Assay Substrate (Promega) according to the manufacturer’s recommendations.

### 2.14 Cell viability assay

The viability of cpd C, cpd F, and cpd G-treated Calu-3 cells was assessed using the CellTiter-Glo 2.0 Cell Viability Assay (Promega) according to the manufacturer’s instructions. Briefly, cells were seeded in 96-well plates and treated with the indicated concentrations of compound. After 48 h, cells were lysed and the luminescence signal was measured using a BioTek multi-well plate reader. Viability was calculated in relation to untreated cells and reported as percent of vehicle control.

### 2.15 Statistics

**FIGURE 1:**
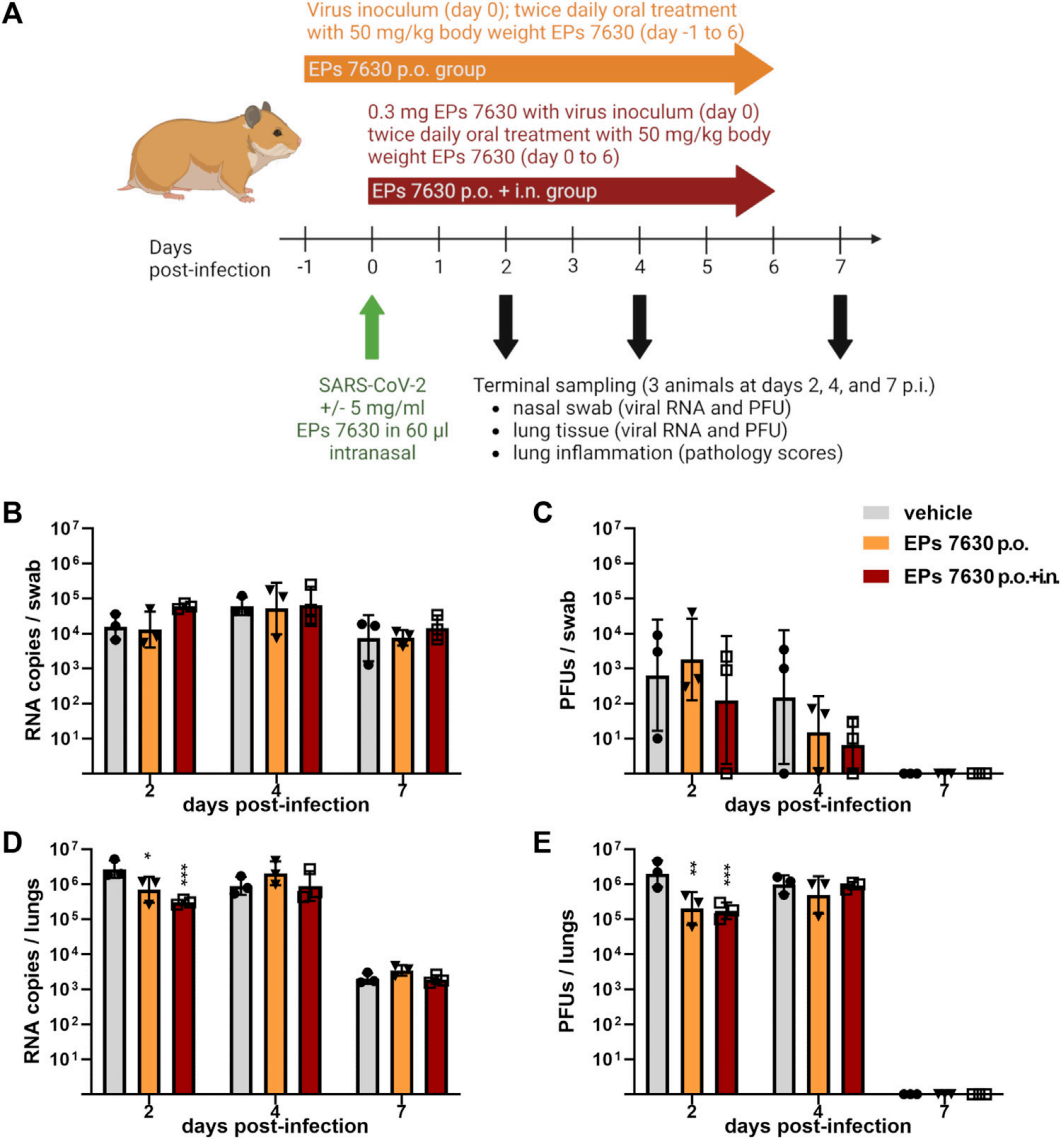
EPs^®^ 7630 has limited effects on virus replication *in vivo*. **(A)** Schematic treatment overview. Hamsters received *Pelargonium sidoides* root extract either orally (p.o.) or both orally and intranasally (p.o. + i. n.). On day 0 animals were infected with 10^5^ PFU SARS-CoV-2 B.1 variant euthanized on day 2, 4 and 7 after infection. **(B)** Genomic viral RNA copies and **(C)** PFU per swab. **(D)** Genomic viral RNA copies and **(E)** PFU in homogenized lung tissue. Statistical significance is indicated by (*) as determined by two-way ANOVA of the log-transformed data with Dunnett’s multiple comparison test. Asterisks are shown only for significantly different data sets. (*) = *p* < 0.05; (**) = *p* < 0.01; (***) = *p* < 0.001.

For the purpose of determining whether treatments resulted in statistically significant outcomes compared to vehicle-treated controls, two-way ANOVAs were performed with Dunnett’s multiple comparison test (Figure 1, Figure 3, Figure 6, Figure 7, Figure 8 and Supplementary Figure S2). To compare the growth kinetics of multiple SARS-CoV-2 variants, two-way ANOVAs were performed with Tukey’s multiple comparison test (Figures 4B, Figure 5). To obtain a statistically conservative indicator of whether SARS-CoV-2 induced cytokine release was reduced upon EPs^®^ 7630 treatment, paired t-tests were performed (Figure 4C). Virus replication data was always log10-transformed prior to statistical analysis (Figures 1, Figure 4B, Figure 5).

## 3 Results

### 3.1 EPs^®^ 7630 exhibits antiviral effects and reduces pathology *in vivo*

Our previous data showed antiviral effects of EPs^®^ 7630 against SARS-CoV-2 (Bavarian strain, B.1) in human lung cells. To assess the effect of EPs^®^ 7630 on SARS-CoV-2 B.1 infection *in vivo*, we employed a previously established Syrian hamster model (Osterrieder et al., 2020; Nouailles et al., 2021). Two experimental groups were used. For the first experimental group, a prophylactic pre-treatment strategy was used, such that the hamsters received oral treatment twice daily at a dose of 50 mg/kg body weight EPs^**®**^ 7630, beginning 24 h prior to infection. For the second experimental group, a combined oral plus intranasal therapeutic strategy was used, such that the hamsters received EPs^**®**^ 7630 at 5 mg/kg body weight intranasally once, together with the virus inoculum in addition to oral treatment twice daily at a dose of 50 mg/kg body weight EPs^**®**^ 7630, beginning at the time of infection. The control group was infected and received only vehicle without EPs^**®**^ 7630. A summary of the treatment scheme and sampling times is depicted in Figure 1A.

EPs^**®**^ 7630 showed limited antiviral activity in the hamster model. Swabs were taken to monitor virus replication in the upper respiratory tract. No significant antiviral effects were observed in the upper respiratory tract (Figures 1B,C). Lung tissue was also examined to evaluate SARS-CoV-2 replication in the lower respiratory tract (Figures 1D,E). Both oral only and oral plus i. n. treatment groups exhibited an approximately 10-fold reduction in SARS-CoV-2 PFU at 2 days post-infection (dpi) compared to vehicle-treated controls, however this difference was not apparent at 4 dpi (Figure 1E). Both the oral plus i. n. treatment group and the oral only group exhibited a statistically significant reduction in both SARS-CoV-2 PFU and RNA in the lower respiratory tract at 2 dpi, with slightly more prominent effects seen within the oral plus i. n. group (Figures 1D,E). Overall, the data suggest that EPs^®^ 7630 delays the onset of virus replication in the lower respiratory tract.

**FIGURE 2:**
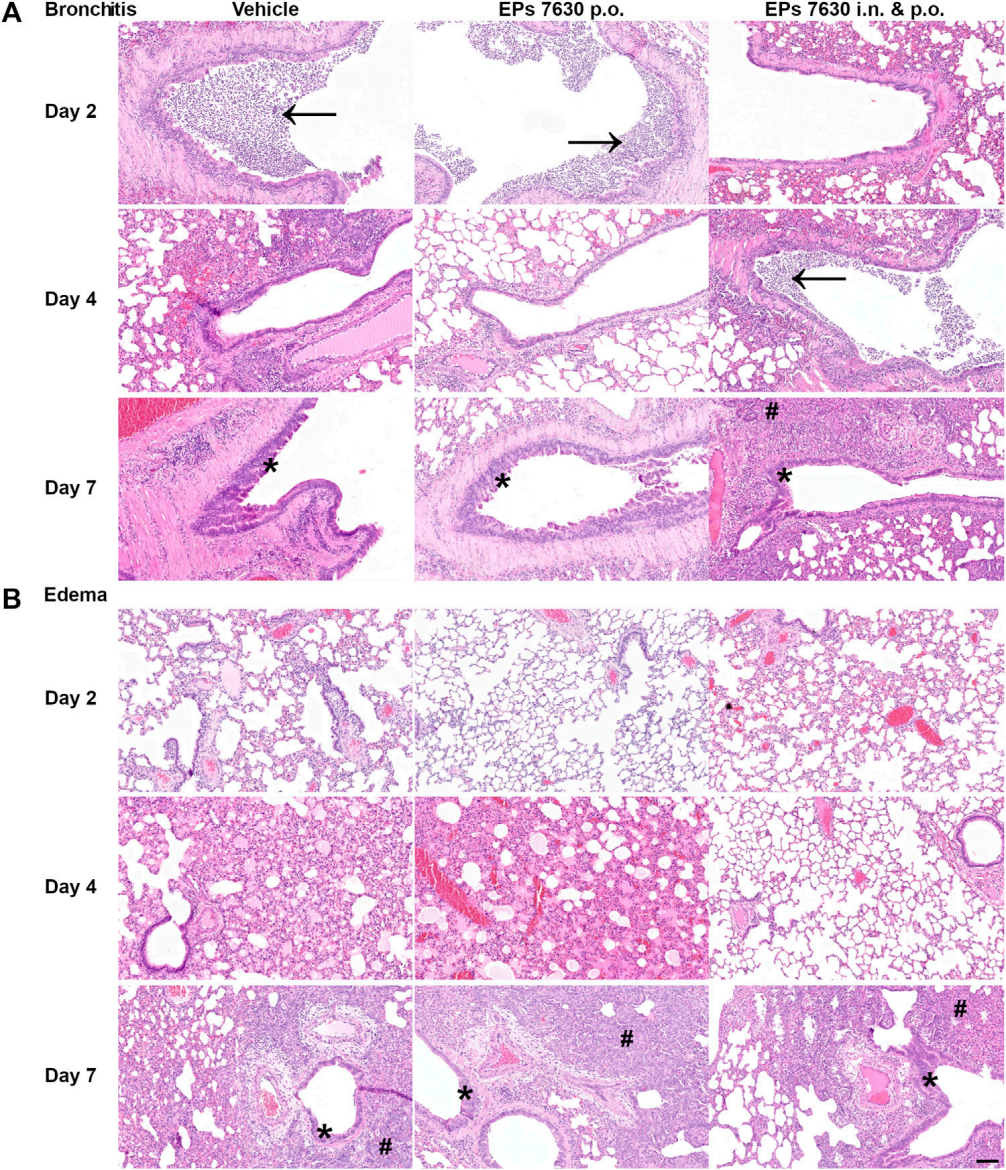
Histopathology reveals significant effects of EPs^®^ 7630 on lungs of SARS-CoV-2-infected hamsters. Histopathology of representative hematoxylin-and-eosin-stained, paraffin-embedded left lungs comparing the three groups (vehicle, EPs^®^ 7630 p. o., EPs^®^ 7630 i. n. and p. o.) in parameters bronchitis and edema. **(A)** Severe bronchitis in the control group (vehicle, left column), indicated by large amounts of neutrophils in the bronchial lumen (arrow) as well as bronchial epithelial cell necrosis. Delayed onset of bronchitis in the EPs^®^ 7630 i. n. and p. o. group (right column) with occurrence of neutrophil infiltration in bronchial lumina (arrow) by day 4 of infection. All groups show proliferative regenerative change of the bronchial epithelium with bronchial epithelial hyperplasia in the late stage (asterisk) by day 7 of infection (bottom row). **(B)** Perivascular and alveolar edema formation less prominent in the EPs^®^ 7630 i. n. and p. o. group in comparison to vehicle and EPs^®^ 7630 p. o. group (middle row, day 4 of infection). Prominent regenerative change in all groups during the late stage (day 7 of infection) with strong pneumocyte type 2 hyperplasia (hash symbol) (bottom row). Scale bar 100 µm for all pictures.

Lung histopathology (hematoxylin-and-eosin-stained, paraffin-embedded left lungs) was performed to determine whether EPs^®^ 7630 treatment also resulted in a change in COVID-19 pathology (Figure 2). Representative sections of selected observed pathologies (bronchitis and edema) were compared between the three groups (vehicle, EPs^®^ 7630 p. o., EPs^®^ 7630 i. n. and p. o.) (Figure 2). Bronchitis and bronchial epithelial cell necrosis were severe in the control group, with a delayed onset of onset of bronchitis in the EPs^®^ 7630 i. n. and p. o. group (Figure 2A). In comparison to vehicle and EPs^®^ 7630 p. o. group, perivascular and alveolar edema formation was less apparent in the EPs^®^ 7630 i. n. and p. o. group (Figure 2B). Regenerative changes with strong pneumocyte type 2 and bronchial epithelial hyperplasia changes were noted in all groups during the late stage of infection.

**FIGURE 3:**
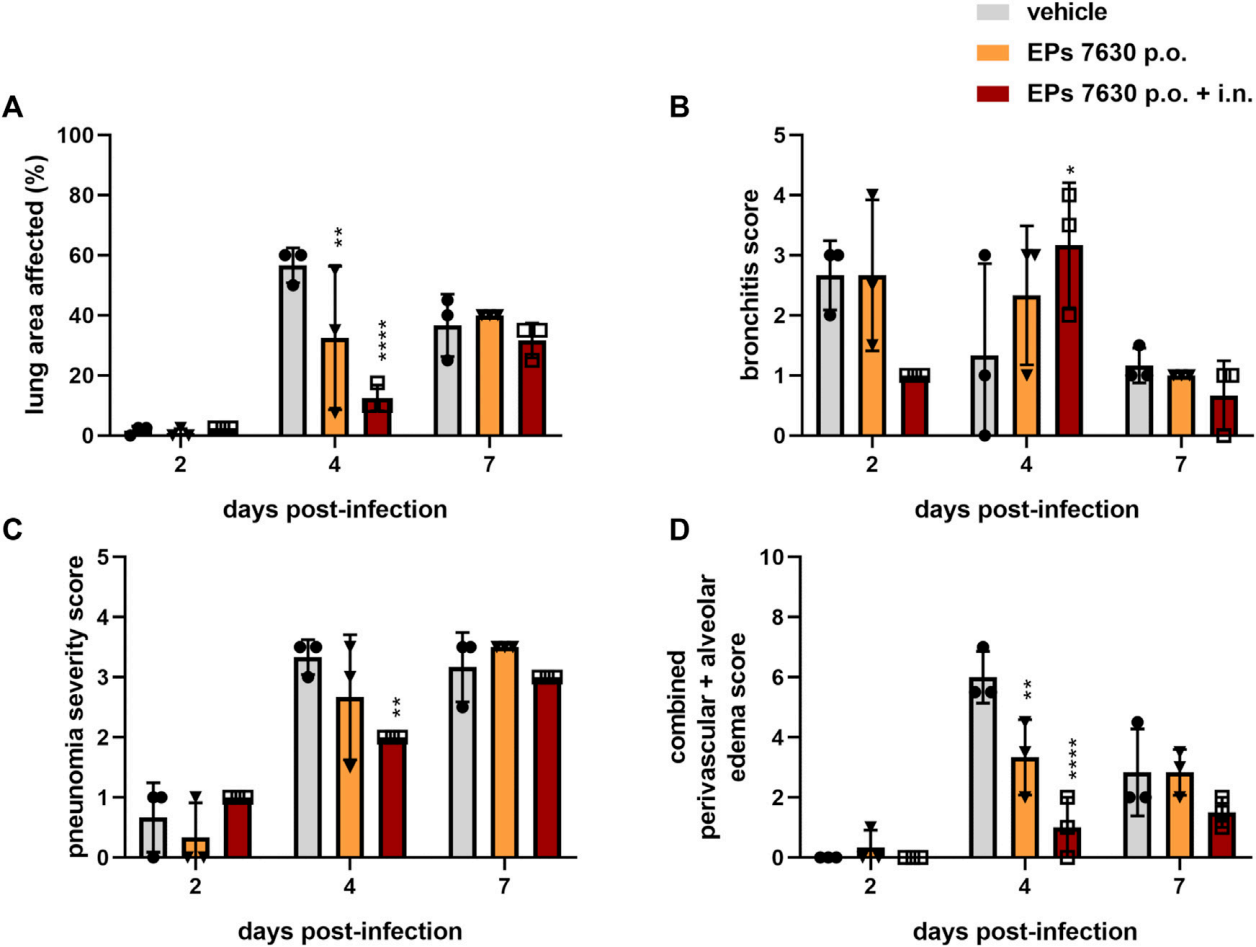
EPs^®^ 7630 delays bronchiolitis and limits lung edema in SARS-CoV-2-infected hamsters. Hamsters received *Pelargonium sidoides* root extract either orally (p.o.) or both orally and intranasally (p.o. + i. n.) as described in Figure 1. **(A)** Approximate lung area affected by inflammatory damage in percentage per group and time point. **(B–D)** Semi-quantitative scoring of pneumonia severity **(B)** bronchitis **(C)** and Edema (perivascular and alveolar) **(D)** for all groups and respective time points. Statistical significance is indicated by (*) as determined by two-way ANOVA of the data with Dunnett’s multiple comparison test. Asterisks are shown only for significantly different data sets. (*) = *p* < 0.05; (**) = *p* < 0.01; (***) = *p* < 0.001; (****) = *p* < 0.0001.

Specifically, semi-quantitative scoring was performed on representative hematoxylin-and-eosin-stained, paraffin-embedded left lung tissue samples (Figure 3). EPs^®^ 7630-treated hamsters had significantly lower amounts of lung area exhibiting signs of disease pathology at 4 dpi than vehicle-treated control hamsters (Figure 3A). This difference was no longer apparent at 7 dpi (Figure 3A). EPs^®^ 7630-treated hamsters also exhibited delayed bronchitis, such that vehicle-treated hamsters had peak bronchitis scores at 2 dpi and EPs^®^ 7630-treated hamsters exhibited similar peak scores at 4 dpi (Figure 3B). P.o. and i. n. EPs^**®**^ 7630-treated hamsters had a significantly lower pneumonia score at 4 dpi, but comparable scores to controls at 7 dpi, suggesting an EPs^®^ 7630-mediated delay of pneumonia onset (Figure 3C). Finally, EPs^®^ 7630-treated hamsters showed markedly reduced lung edema compared to vehicle-treated controls at 4 dpi. This was most apparent for the combined oral plus i. n. treatment group, which continued to exhibit less edema than vehicle-treated controls at 7 dpi (Figure 3D). In summary, EPs^®^ 7630 treatment, especially when combined with intranasal therapy directly with the SARS-CoV-2 inoculum, resulted in delayed COVID-19 pathology, consistent with immunomodulatory activity early during the course of infection.

### 3.2 EPs^®^ 7630 does not have significant antiviral activity against SARS-CoV-2 B.1, but exhibits strong anti-inflammatory effects in human bronchial airway epithelial cells

**FIGURE 4:**
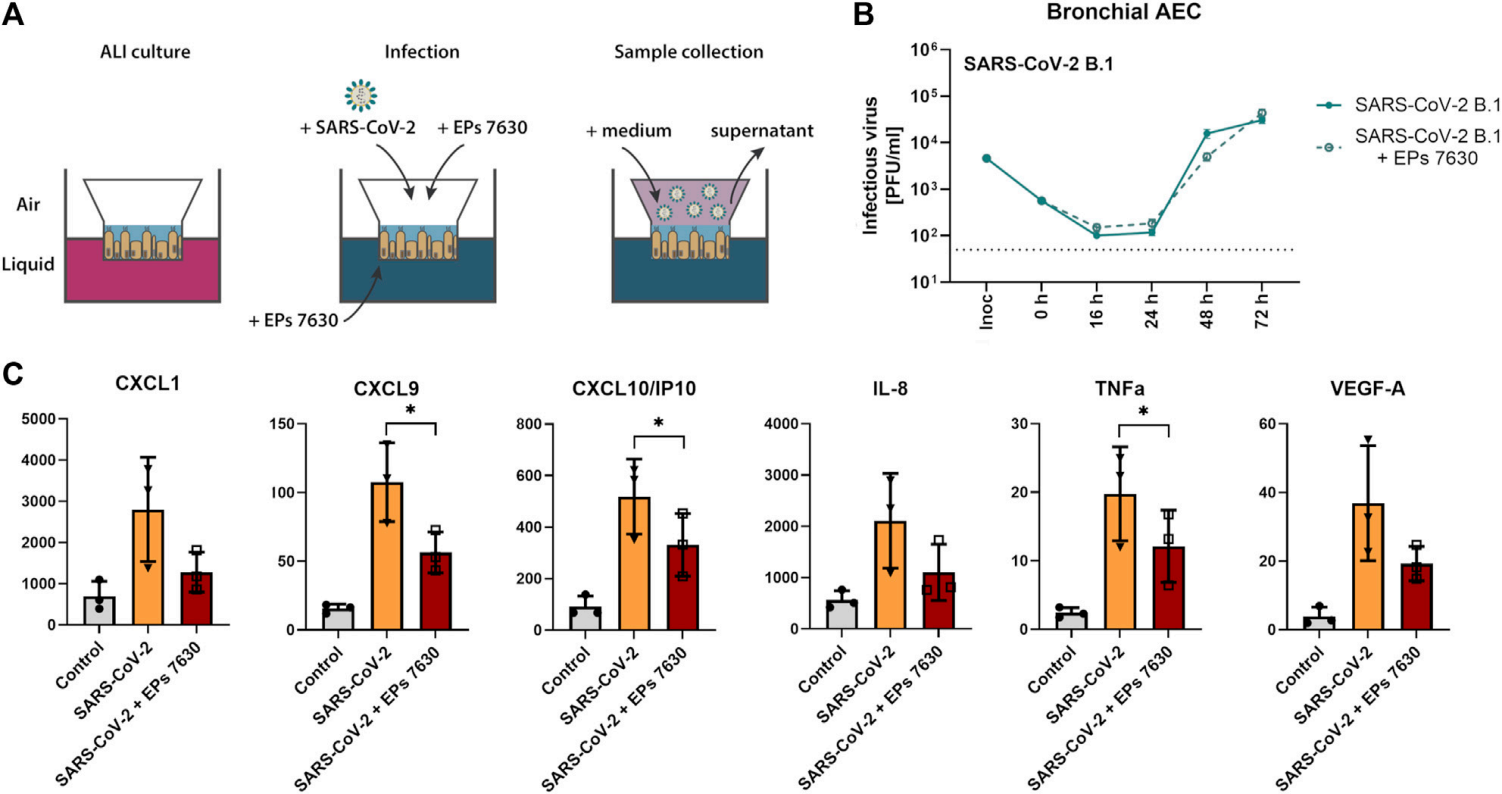
SARS-CoV-2 (B.1) propagation and inflammatory cytokine expression in human bronchial airway epithelial cells (bAEC). **(A)** Bronchial AEC were inoculated with SARS-CoV-2 (MOI = 0.005) with and without EPs^®^ 7630 (100 μg/mL) treatment at 37°C for 2 h. For sample collection, the apical side of the bAEC was incubated with 250 µL Mucilair medium for 20 min, which was subsequently removed and frozen at −80°C until analysis. Supernatants were analyzed by plaque assays between 0 and 72 h post-infection **(B)** or at 24 h post-infection using the Human Cytokine/Chemokine/Growth Factor Multiplex Assay (Merck Millipore) with the Luminex MAGPIX System according to the manufacturer’s instructions **(C)**. Data are derived from *n* = 3 biological samples. Cell culture medium was used as the vehicle control. No statistical significance was observed for **(B)**, as determined by two-way ANOVA with Tukey’s multiple comparison test on log-transformed data. Statistical significance for **(C)** was determined by paired t-tests. Asterisks are shown only for significantly different data sets. (*) = *p* < 0.05. ALI = air liquid interface.

Our previous investigation of EPs^®^ 7630 indicated that it had an antiviral effect in Calu-3 cells (Papies et al., 2021). To obtain more evidence about whether EPs^®^ 7630 may be a suitable antiviral drug in the context of human SARS-CoV-2 infection, experiments were performed in human bAEC. EPs^®^ 7630 (100 μg/mL) treatment was performed in an air-liquid interface setup, as depicted in Figure 4A. Plaque assays were performed on supernatants sampled at 0, 16, 24, 48, and 72 h after SARS-CoV-2 infection. EPs^®^ 7630 treatment was associated with a small decrease in SARS-CoV-2 PFU at 48 h post-infection, yet peak viral titers were similar between treated and untreated controls at 72 h post-infection (Figure 4B). To evaluate whether EPs^®^ 7630 treatment has potential implications for human disease pathology, cytokines in bAEC supernatant were quantified at 24 h post-infection. EPs^®^ 7630 treatment caused a statistically significant reduction in inflammatory cytokines, notably CXCL9, CXCL10/IP-10, and TNF-α (Figure 4C). Although variance was greater, the release of chemokines CXCL1 and IL-8, as well as VEGF-A was also considerably reduced due to EPs^®^ 7630 treatment (Figure 4C). Overall, the bAEC data show that EPs^®^ 7630 has a limited antiviral effect against SARS-CoV-2 B.1, but a pronounced immunomodulatory effect that may help prevent excessive pro-inflammatory cytokine release and disease pathology.

### 3.3 EPs^®^ 7630 exhibits antiviral activity against SARS-CoV-2 variants Delta and Omicron in human airway epithelial cells

Based on previous data suggesting that EPs^®^ 7630 primarily exerts its antiviral effects by limiting SARS-CoV-2 entry (Papies et al., 2021), we hypothesized that EPs^®^ 7630 may differentially affect SARS-CoV-2 variant Omicron, which apparently exhibits a preference for TMPRSS2-independent late endosomal entry instead of plasma membrane fusion compared to prior variants (Meng et al., 2022; Willett et al., 2022). To evaluate whether EPs^®^ 7630 differentially affects viruses with distinct entry routes, we performed infection experiments with SARS-CoV-2 variants Omicron BA.2 and Delta AY.4 for comparison. Additionally, we infected nasal AEC (nAEC) and bAEC to model both the upper and lower respiratory tract. Viral PFU were quantified at 0, 16, 24, 48, and 72 h post-infection.

**FIGURE 5:**
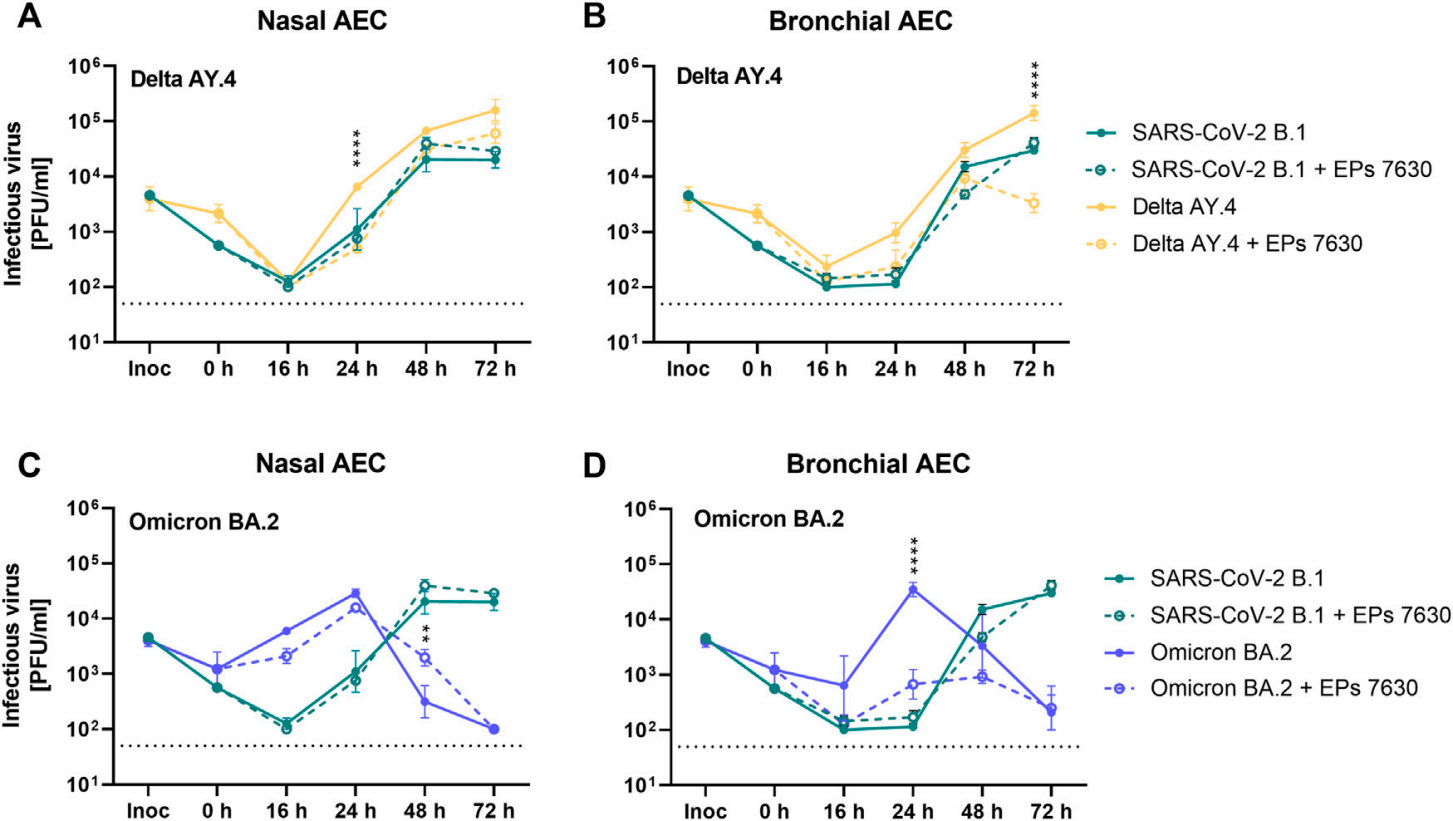
Enhanced inhibition of SARS-CoV-2 variant BA.2 by EPs^®^ 7630 in human bronchial airway epithelial cells (bAEC). Nasal AEC **(A, C)** and bAEC **(B, D)** were inoculated with SARS-CoV-2 variant AY.4 **(A, B)** or BA.2 **(C, D)** using an MOI of 0.005 with and with EPs^®^ 7630 (100 μg/mL) treatment at 37°C for 2 h. For sample collection, the apical side of the AEC was incubated with 250 µL Mucilair medium for 20 min, which was subsequently removed and frozen at −80°C until analysis. Supernatants were analyzed by plaque assays between 0 and 72 h post-infection. Data are derived from *n* = 3 biological samples. Variant growth kinetics are shown in parallel with the B.1 growth kinetics previously depicted in Figure 4B. Cell culture medium was used as the vehicle control. Statistical significance was determined by two-way ANOVA with Tukey’s multiple comparison test on log-transformed data. Asterisks are shown when EPs^®^ 7630 treatment resulted in significantly different levels of Delta or Omicron PFU for given timepoints. (*) = *p* < 0.05; (**) = *p* < 0.01; (***) = *p* < 0.001; (****) = *p* < 0.0001.

SARS-CoV-2 Delta had slightly higher replication efficiency than SARS-CoV-2 B.1 in both nAEC and bAEC models (Figures 5A,B, B.1 data shown from Figure 4B). In nAECs, EPs^®^ 7630 treatment significantly reduced SARS-CoV-2 Delta replication at 24 h post-infection, such that SARS-CoV-2 Delta infection with EPs^®^ 7630 treatment closely resembled the kinetics of infection for SARS-CoV-2 B.1 without EPs^®^ 7630 treatment (Figure 5A). In bAECs, EPs^®^ 7630 resulted in an anomalously low SARS-CoV-2 Delta titer at 72 h post-infection, representing an approximately 100-fold reduction in PFU (Figure 5B). Overall, EPs^®^ 7630 seemed to have a slightly more potent effect against SARS-CoV-2 Delta than against the B.1 strain.

In contrast to SARS-CoV-2 B.1 and SARS-CoV-2 Delta strains, SARS-CoV-2 Omicron exhibited distinct replication kinetics (Figures 5C,D). Whereas previous strains had peak titers at 72 h post-infection, SARS-CoV-2 Omicron replicated more quickly and exhibited a peak titer at 24 h post-infection. EPs^®^ 7630 treatment resulted in a small decrease in SARS-CoV-2 replication in nAECs at 16 h post-infection, consistent with delayed virus replication (Figure 5C). EPs^®^ 7630 exhibited a moderate antiviral effect in bAECs against Omicron, resulting in an approximately 100-fold PFU reduction at 24 h post-infection, with reductions also visible at 16 h and 48 h post-infection (Figure 5D, B.1 data shown from Figure 4B). Thus, SARS-CoV-2 Omicron appeared to have increased susceptibility to EPs^®^ 7630 treatment, compared to either SARS-CoV-2 B.1 or SARS-CoV-2 Delta.

### 3.4 EPs^®^ 7630 partially inhibits SARS-CoV-2 S-mediated entry, with stronger effects against Omicron BA.2 S

**FIGURE 6:**
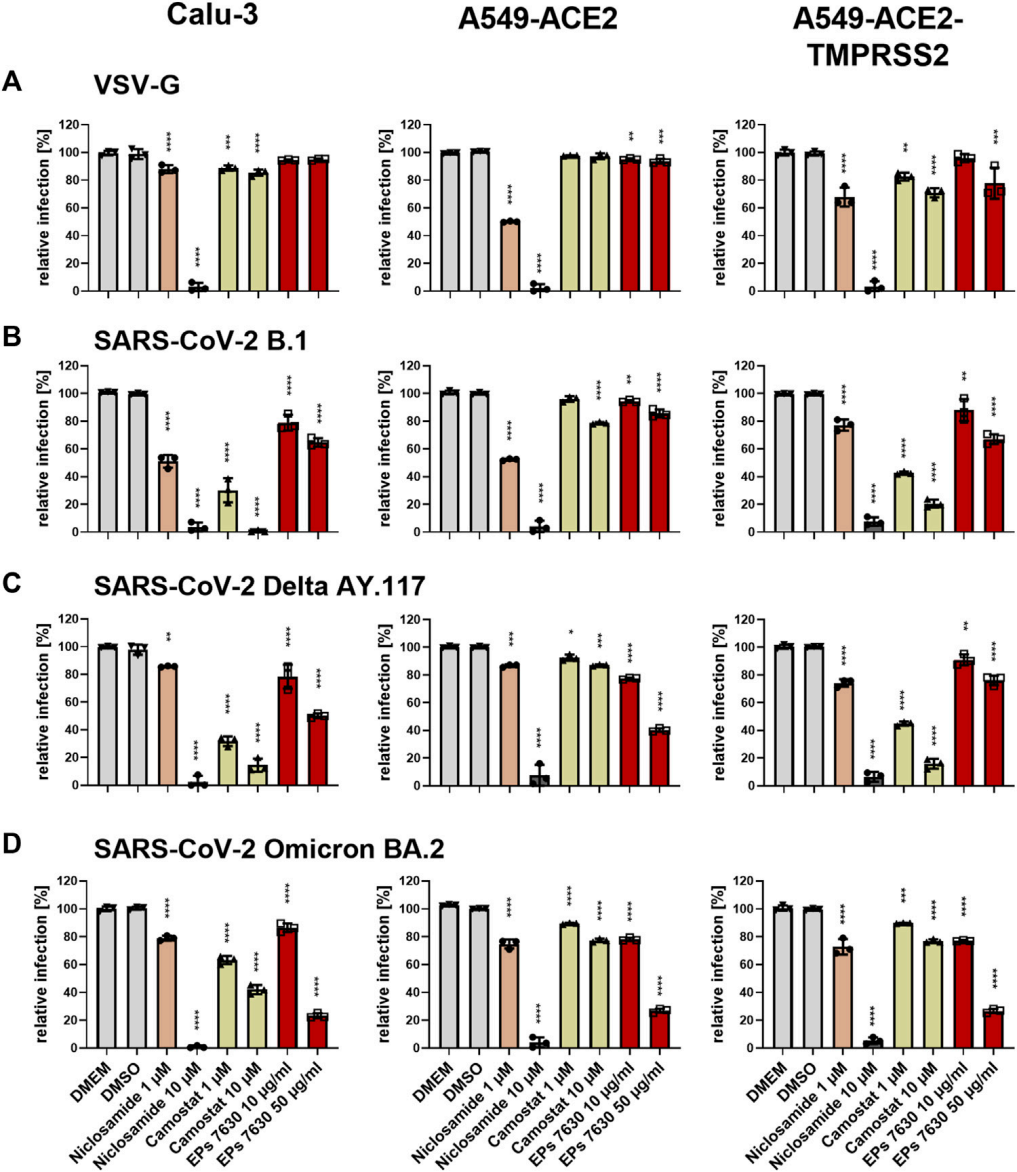
Differential entry inhibition of SARS-CoV-2 variants by EPs^®^ 7630. All cells were pre-treated with the indicated compounds for 2 h pre-infection at 37°C. Infection of Calu-3, A549-ACE2, and A549-ACE2/TMPRSS2 cells with VSV-G as control **(A)**, SARS-CoV-2-S VSVpp (SARS-CoV-2-S) from B.1 **(B)**, variant Delta AY.117 **(C)**, or variant Omicron BA.2 **(D)** was done in the presence of compounds for 30 min at 4°C at 300 *g* followed by 1-h incubation at 37°C. The medium was then replaced by DMEM containing the indicated compounds. DMSO was additionally used as a vehicle control. As positive controls, we applied 1 and 10 µM niclosamide (pH-dependent endosomal entry inhibitor) and 1 and 10 µM camostat mesylate (TMPRSS2 inhibitor). Cell lysates were prepared after 24 h and the luciferase signal was measured using a multi-mode 96-well plate reader. Statistical significance is indicated by (*) as determined by two-way ANOVA of the data with Dunnett’s multiple comparison test. Asterisks are shown only for significantly different data sets. (*) = *p* < 0.05; (**) = *p* < 0.01; (***) = *p* < 0.001; (****) = *p* < 0.0001.

To further clarify a putative mechanism by which EPs^®^ 7630 inhibits SARS-CoV-2 infection, we investigated whether EPs^®^ 7630 treatment had differential effects on SARS-CoV-2 S-mediated entry in several cell lines: Calu-3, A549 stably transfected with ACE2, and A549 stably transfected with ACE2 and TMPRSS2 (Widera et al., 2021). Camostat mesylate is a serine protease inhibitor and is known to inhibit TMPRSS2-mediated processing of the SARS-CoV-2 S protein to promote virus entry (Hoffmann et al., 2020). Niclosamide is a protonophore that has been previously shown to inhibit SARS-CoV-2 infection, and was used as a positive control for antiviral activity (Gassen et al., 2021) and pH-dependent endosomal entry inhibition (Jurgeit et al., 2012). A VSVpp entry assay was performed using VSV-G, B.1, Delta, and Omicron S. DMSO, niclosamide, camostat mesylate, or EPs^®^ 7630 were administered as a pre-treatment for 2 h prior to infection and present in VSVpp inoculum. Readout was performed by quantifying luciferase activity of the cell lysates at 24 h post-infection. As expected, low pH-dependent endosomal entry of VSV-G carrying VSVpp was strongly blocked by niclosamide but moderately affected by camostat mesylate (Figure 6A). Camostat potently inhibited the entry of B.1 and Delta S VSVpp in TMPRSS2-positive cell lines, Calu-3 and A549-ACE2-TMPRSS2. In contrast, camostat mesylate did not have a strong effect in the TMPRSS2-negative cell line, A549-ACE2, exhibiting <25% inhibition (Figures 6B,C). Consistent with other reports that Omicron preferentially enters through endosomal pathway and does not depend on TMPRSS2 (Pia and Rowland-Jones, 2022), we found that TMPRSS2 did not promote SARS-CoV-2 Omicron VSVpp entry, such that the effect of camostat mesylate was comparable between A549-ACE2 and A549-ACE2-TMPRSS2 cell lines (Figure 6D). Whereas 1 μM camostat mesylate was sufficient for >50% reduction of B.1 and Delta S-based VSVpp entry in TMPRSS2-positive cell lines, Omicron S exhibited decreased sensitivity to camostat mesylate, consistent with a low level of TMPRSS2-dependent entry inhibition.

In direct contrast to camostat mesylate, EPs^®^ 7630 resulted in more potent inhibition of Omicron S-mediated entry and weaker inhibition of B.1 and Delta S-mediated entry. Specifically, 50 μg/mL EPs^®^ 7630 was sufficient to reduce Omicron S-mediated entry by approximately 75%, whereas B.1 (Figure 6B) and Delta S (Figure 6C) mediated entry was only slightly limited with inhibition in the range of 15%–65%. Although dose-dependent virus entry inhibition by EPs^®^ 7630 was observed in all cases, the differences between S variants was less apparent at the lower dose of 10 μg/mL, where only a slight 5%–25% reduction in VSVpp entry was observed.

### 3.5 (−)-Epigallocatechin, (−)-epigallocatechin gallate, and (+)-taxifolin partially inhibit SARS-CoV-2 propagation

**FIGURE 7:**
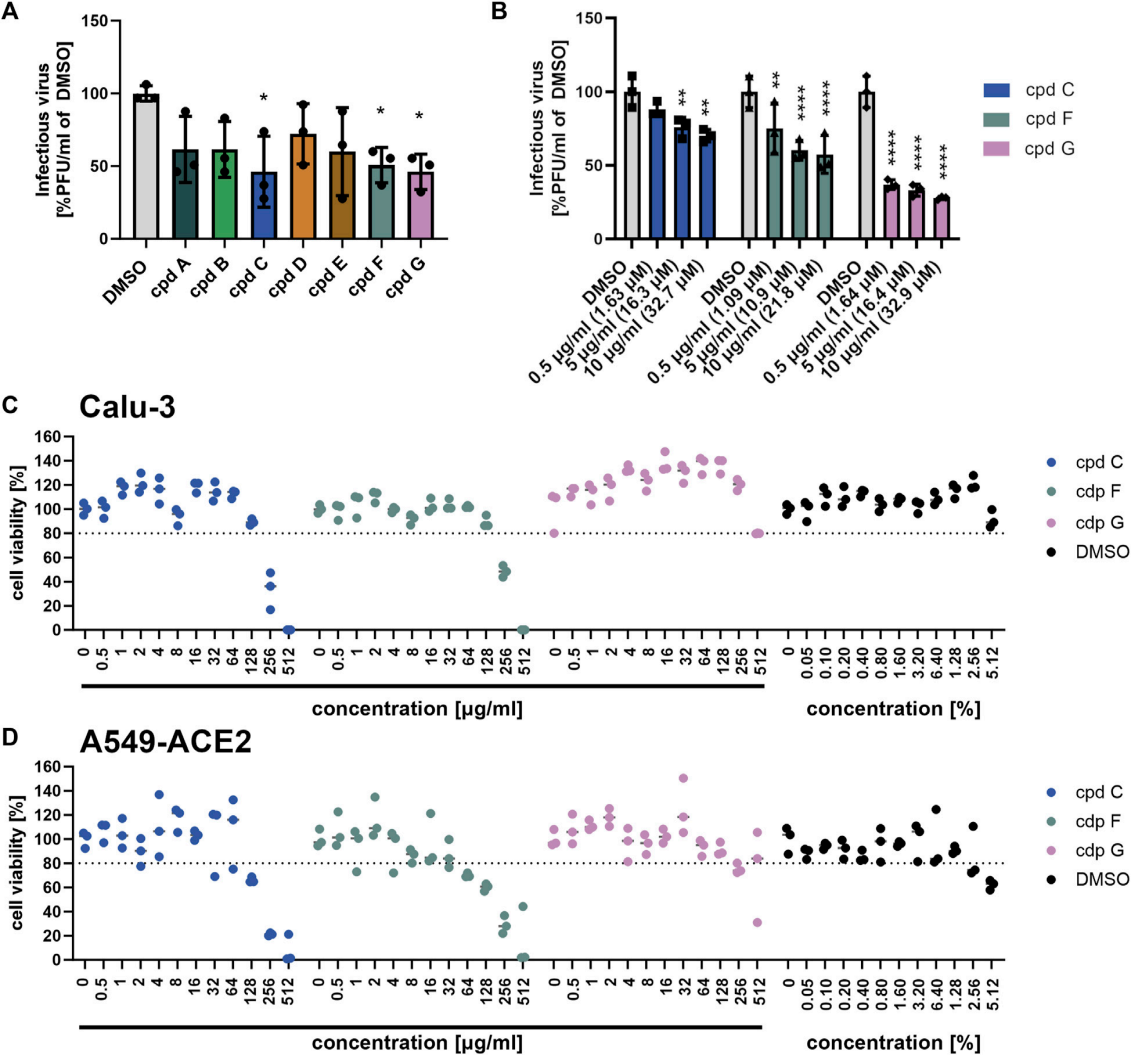
Epigallocatechin, epigallocatechin gallate, and taxifolin inhibit SARS-CoV-2 B.1 propagation dose dependently. **(A-B)** Calu-3 cells were infected with SARS-CoV-2 (MOI = 0.0005) and treated with 7 defined low molecular weight constituents of EPs^®^ 7630 using 10 μg/mL, as well as DMSO as a vehicle control **(A)**, and additionally for cpd C (epigallocatechin), cpd F (epigallocatechin gallate), and cpd G (taxifolin) in a dilution series of 0.5–10 μg/mL **(B)**. Virus-containing supernatants were collected 24 h post-infection and viral titers were determined as PFU/mL by plaque titration assay. For both Calu-3 **(C)** and A549-ACE2 cells **(D)**, compound toxicity was evaluated by performing a CellTiter Glo assay in a dilution range of 0.5–512 μg/mL for each compound and 0.5%–5.12% DMSO (to evaluate vehicle toxicity) at 24 h post-infection. Data are derived from *n* = 3 biological samples. Statistical significance is indicated by (*) as determined by two-way ANOVA of the data with Dunnett’s multiple comparison test. Asterisks are shown only for significantly different data sets. (*) = *p* < 0.05; (**) = *p* < 0.01; (***) = *p* < 0.001; (****) = *p* < 0.0001.

To further investigate which constituents of EPs^**®**^ 7630 might be involved in inhibition of SARS-CoV-2 entry, we performed infection experiments with seven small molecules that are known or putative constituents in EPs^**®**^ 7630 (Table 1). The seven substances were administered together with the viral inocula and evaluated for their antiviral effect on SARS-CoV-2 B.1 in Calu-3 cells at a concentration of 10 μg/mL (Figure 7A). The constituents (−)-epigallocatechin (cpd C) and (+)-taxifolin (cpd G), and the putative constituent, (−)-epigallocatechin gallate (cpd F), resulted in a statistically significant decrease in SARS-CoV-2 PFUs at 24 h post-infection and were thus selected for further investigation. Cpd C, cpd F, and cpd G were tested for dose dependence at concentrations of 0.5, 5.0, and 10.0 μg/mL using the same treatment scheme as described above. Dose-dependent antiviral activity was observed for all three substances. At 10 μg/mL, SARS-CoV-2 PFU exhibited a mean reduction of 29.8% for cpd C, 42.6% for cpd F, and 72.2% for cpd G (Figure 7B) at non-toxic concentrations (Figures 7C,D).

### 3.6 Entry inhibition appears to be an important mechanism of antiviral activity for the epigallocatechins, but not for taxifolin

**FIGURE 8:**
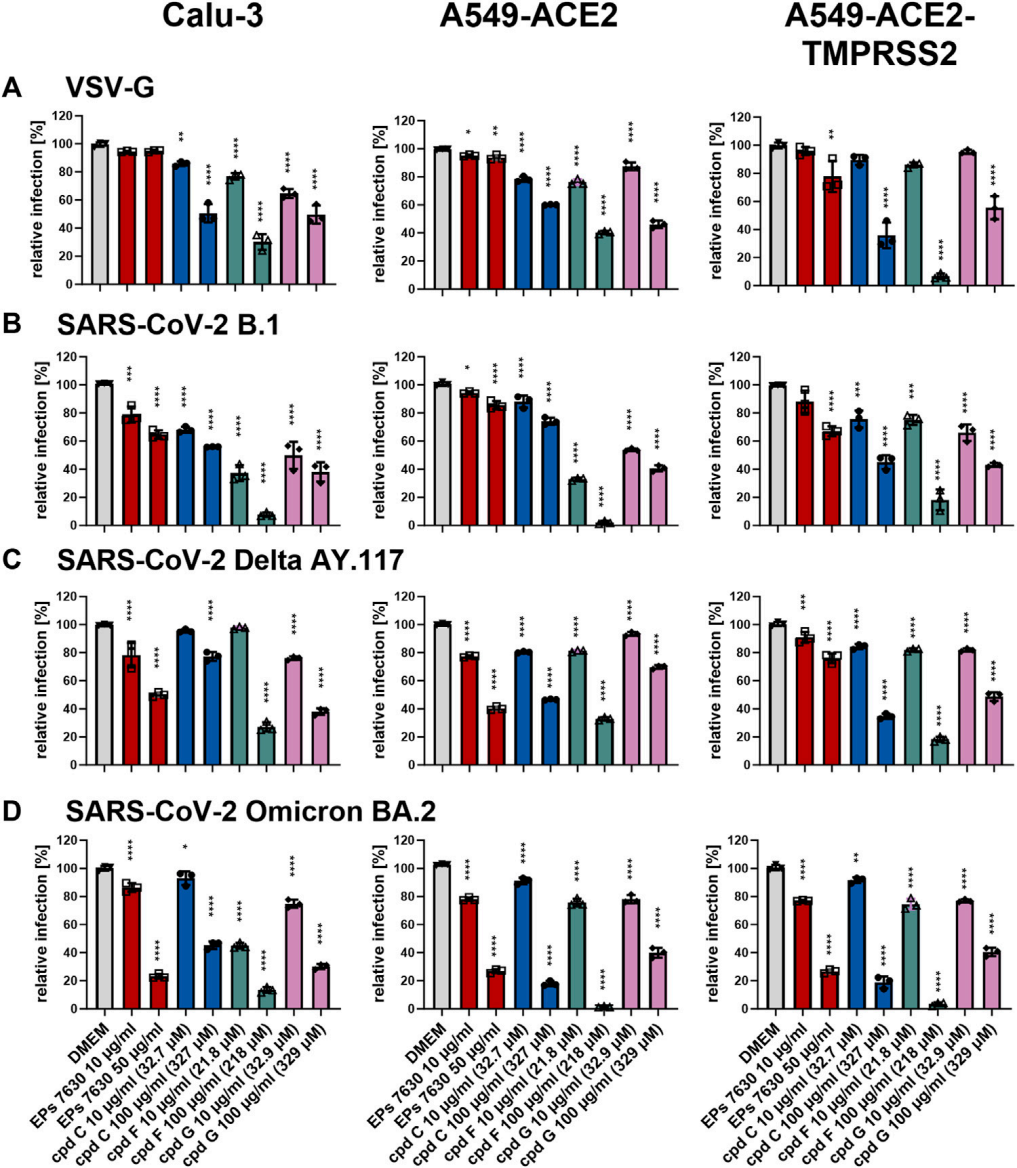
Epigallocatechin and epigallocatechin gallate inhibit predominantly endosomal-mediated SARS-CoV-2 entry showing enhanced activities for B.1 and Omicron BA.2. All cells were pre-treated with the indicated compounds for 2 h pre-infection at 37°C. Infection of Calu-3, A549-ACE2, and A549-ACE2-TMPRSS2 cells with VSV-G as control **(A)**, SARS-CoV-2-S VSVpp (SARS-CoV-2-S) from B.1 **(B)**, variant Delta AY.117 **(C)**, or variant Omicron BA.2 **(D)** was done in the presence of compounds for 30 min at 4°C at 300 *g* followed by 1-h incubation at 37°C. The medium was then replaced by DMEM containing the indicated compounds. DMSO was additionally used as a vehicle control. For comparison, the EPs^®^ 7630 data from Figure 6 were included as all experiments were done in parallel. Cell lysates were prepared after 24 h and the luciferase signal was measured using a multi-mode 96-well plate reader. Statistical significance is indicated by (*) as determined by two-way ANOVA of the data with Dunnett’s multiple comparison test. Asterisks are shown only for significantly different data sets. (*) = *p* < 0.05; (**) = *p* < 0.01; (***) = *p* < 0.001; (****) = *p* < 0.0001.

To determine whether the antiviral effects of cpd C, cpd F, or cpd G are based on entry inhibition, a VSVpp entry assay was performed using VSV-G (for comparison, Figure 8A), B.1 (Figure 8B), Delta (Figure 8C), and Omicron (Figure 8D) S proteins. Vehicle control (DMEM), cpd C, cpd F, and cpd G were administered as a pre-treatment for 2 h prior to infection and present in VSVpp inoculum, and readout was performed by quantifying luciferase activity of the cell lysates at 24 h post-infection. For direct comparison, EPs^**®**^ 7630 data from Figure 6 is also shown in this Figure (experiments were done in parallel). All three compounds exhibited dose-dependent entry inhibition. Depending on the cell type, at 100 μg/mL cpd C had a 9.8%–56.1% stronger effect against Omicron S-mediated entry (Figure 8D) compared to B.1 and Delta S proteins (Figures 8B,C). Cpd F was the most potent viral entry inhibitor, as 100 μg/mL resulted in mean reductions of between 66.5% and 98.5%, depending on the variant and cell line. Cpd G showed generally consistent levels of entry inhibition regardless of the S variant, with perhaps slightly greater inhibition of the B.1 S (Figure 8B). Interestingly, at 10 μg/mL cpd G showed more enhanced antiviral activity (see Figures 7A,B) than suggested by its entry inhibition (Figure 8), indicating a possible alternative mechanism of antiviral activity. The effects of 10 μg/mL cpd C, cpd F, and cpd G were also tested against additional SARS-CoV-2 variant S proteins, including VSVpp for Alpha B.1.1.7, Beta B.1.351, and Gamma P.1 (Supplementary Figure S2). At this concentration, Alpha, Beta, and Gamma were all inhibited comparably to other variant strains, Delta and Omicron (Figures 7C,D).

## 4 Discussion

This study presents an in-depth investigation into the antiviral and immunomodulatory properties of EPs^**®**^ 7630 with regards to SARS-CoV-2 infection. Our *in vivo* infections of Syrian hamsters show that EPs^®^ 7630 has statistically significant antiviral effects at 2 days post-infection, while our *in vitro* work shows substantial immunomodulatory properties of EPs^**®**^ 7630. Nevertheless, determining the optimal dosage, route, and timing of EPs^**®**^ 7630 administration remains a challenging obstacle to clinical implementation of EPs^**®**^ 7630 as an anti-SARS-CoV-2 therapeutic. Specifically, we have demonstrated that direct application of EPs^**®**^ 7630 leads to delayed onset of COVID-19-like pathology *in vivo* (Figure 2) and a reduction in pro-inflammatory cytokine release *in vitro* (Figures 3, Figure 4). Our data further suggest that high local concentrations of EPs^**®**^ 7630 at the site of virus infection contribute to its therapeutic activity. Whereas 50 μg/mL EPs^®^ 7630 resulted in a moderate inhibitory effect against Omicron *in vitro* (Figure 6D), the pharmacokinetics of EPs^®^ 7630 are not sufficiently characterized to determine whether 50 mg/kg body weight p. o. treatment or 0.3 mg i. n. treatment is equivalent to 50 μg/mL. In the group that received only oral treatment, EPs^**®**^ 7630 activity, as determined by histopathology, was less pronounced compared to combined oral plus intranasal treatment (Figures 1–3). This suggests suboptimal systemic exposure, probably due to limited oral bioavailability of some active constituents in EPs^**®**^ 7630 in our hamster model. Overall, our data suggest that EPs^**®**^ 7630 treatment is not able to achieve the strong degree of antiviral activity exhibited by SARS-CoV-2-specific inhibitors like PF-07321332 (Abdelnabi et al., 2022), but that prophylactic or early treatment with EPs^**®**^ 7630 treatment may still have ameliorative effects on COVID-19. Future studies with either daily intranasal or aerosol lung delivery would be interesting to assess the effects of continuous EPs^**®**^ 7630 therapy.

Compared to our previous study, which indicated that EPs^**®**^ 7630 had antiviral IC50 of 1.61 μg/mL against SARS-CoV-2 B.1 infection in Calu-3 cells (Papies et al., 2021), we did not observe a significant antiviral effect against SARS-CoV-2 B.1 in human AECs (Figure 4B, Figure 5). We attribute this difference to the cell culture model that was used. Nevertheless, significant antiviral effects were observed against Delta AY.4 and Omicron BA.2 in both nasal and bronchial AECs, indicating a variant-specific antiviral effect (Figure 5). As it was beyond the scope of this study, it remains an open question to what degree the immunomodulatory effects we observed (Figure 3, Figure 4C) depend on the activity of EPs^**®**^ 7630 as an entry inhibitor (Figure 6). EPs^**®**^ 7630 has shown efficacy as a clinical therapeutic with positive effects on symptom severity in the context of other respiratory infections (Matthys et al., 2003; Chuchalin et al., 2005; Kamin et al., 2010a; Kamin et al., 2010b; Matthys et al., 2010; Riley et al., 2019) as well as studies in bacterial rhinosinusitis patients where treatment of EPs^**®**^ 7630 additionally affected nasal chemokine levels (Perić et al., 2020; Perić et al., 2021). Therefore, it seems plausible that the therapeutic efficacy of EPs^**®**^ 7630 is due to a combination of inhibition of viral entry and direct anti-inflammatory effects. For example, PB125, another polyphenolic plant extract, has been shown to exhibit similar anti-inflammatory properties, presumably via its upregulation of host transcriptional regulator Nrf2 (Hybertson et al., 2019; McCord et al., 2020). Overall, our data are consistent with the theory that some components of EPs^**®**^ 7630 participate in specific host factor interactions, which lead to both the antiviral and immunomodulatory effects that we observe. However, direct interactions with SARS-CoV-2 proteins cannot be excluded.

In addition to EPs^**®**^ 7630, this study also examined selected constituents of EPs^®^ 7630 that were of interest as potential sources of its antiviral activity. (−)-Epigallocatechin, (+)-taxifolin, and the putative constituent (−)-epigallocatechin gallate were identified as exhibiting antiviral effects against SARS-CoV-2 (Figure 7A). Taxifolin had a more pronounced antiviral effect (extrapolated IC50 < 0.5 μg/mL or 1.64 μM, Figure 7B) than would be suggested by its activity as an entry inhibitor (extrapolated IC50 ≈ 50 μg/mL or 164 μM, Figure 8B), indicating that taxifolin possesses antiviral properties independent of its modest effect on virus entry. This post-entry antiviral activity is consistent with a previous study which has shown that taxifolin inhibits the SARS-CoV-2 main protease with an IC50 of 12.94 μM (Zhu et al., 2022). In contrast, epigallocatechin and epigallocatechin gallate exhibited entry inhibition activity consistent with their antiviral effects (Figures 7, Figure 8), suggesting that entry inhibition was their primary mechanism of antiviral activity. IC50 values for entry inhibition were predicted to be between 10 and 100 μg/mL, in the range of approximately 100 μM (Figure 8). Previous reports have suggested that epigallocatechin gallate can disrupt SARS-CoV-2 Spike/ACE2 binding at relatively low concentrations (IC50 = 0.44 μg/mL or 994.6 nM) (Invernizzi et al., 2021), but we did not observe such a strong degree of inhibition in this study. One additional pharmacological property of epigallocatechin gallate is its inhibition of CYP3A4 (Ikarashi et al., 2016; Ikarashi et al., 2017), a mode of action similar to ritonavir-mediated inhibition of CYP3A4 (Sevrioukova and Poulos, 2010). Overall, however, EPs^**®**^ 7630 showed significantly greater antiviral activity than the individual constituents we selected, demonstrating that none of these components can be considered the sole or primary active ingredient of EPs^**®**^ 7630 (Figure 5, Figure 7B). This supports our previous observation that after fractionation of the extract, several fractions, especially those containing medium-sized polymeric proanthocyanidins showed antiviral activity (Papies et al., 2021). Thus, our findings indicate that multiple components of EPs^**®**^ 7630 are biologically active and contribute to its overall effect in either an additive or synergistic fashion.

In this study, we further demonstrate that the mechanism by which EPs^**®**^ 7630, its constituent epigallocatechin, and its putative constituent epigallocatechin gallate block SARS-CoV-2 entry is dependent upon the SARS-CoV-2 S variant. Specifically, EPs^**®**^ 7630, epigallocatechin, and epigallocatechin gallate showed greater entry inhibition against the Omicron BA.2 S compared to B.1 and Delta AY.117 (Figures 6, Figure 8). This corresponds with the increased antiviral activity of EPs^**®**^ 7630, epigallocatechin, and epigallocatechin gallate against Omicron BA.2 (Figures 5, Figure 7). It has previously been reported that Omicron BA.2 preferentially enters host cells via the endosomal pathway as opposed to TMPRSS2-dependent plasma membrane fusion favored by previous variants (Meng et al., 2022; Willett et al., 2022). This change in entry mechanism is facilitated by mutations in the S protein, such as H655Y, that reduce priming by serine proteases while increasing priming by endosomal proteases (Hu et al., 2022). It cannot be distinguished from our data whether EPs^**®**^ 7630, epigallocatechin, and epigallocatechin gallate interact directly with the S protein or with cellular proteins to achieve their entry inhibition activity. Nevertheless, our findings are consistent with the hypothesis that these compounds act by reducing host endocytosis. Indeed, epigallocatechin gallate has previously been reported to slow host endocytosis (Pan et al., 2002). Other polyphenolic plant compounds, such as those found in hop bract extract, have also been shown to interfere with host endocytosis (Morinaga et al., 2005). Future studies aiming to characterize the function of EPs^**®**^ 7630 should identify specific EPs^**®**^ 7630-modulated pathways or interaction partners to better clarify its cellular targets.

## Data Availability

The original contributions presented in the study are included in the article/Supplementary Material, further inquiries can be directed to the corresponding author.
