# Supplementary figures and images for: In vitro and in vivo effects of Pelargonium sidoides DC. root extract EPs® 7630 and selected constituents against SARS-CoV-2 B.1, Delta AY.4/AY.117 and Omicron BA.2
Source: Front Pharmacol. 2023 Jul 26;14:1214351. doi: 10.3389/fphar.2023.1214351 (PMC10410074; doi:10.3389/fphar.2023.1214351)

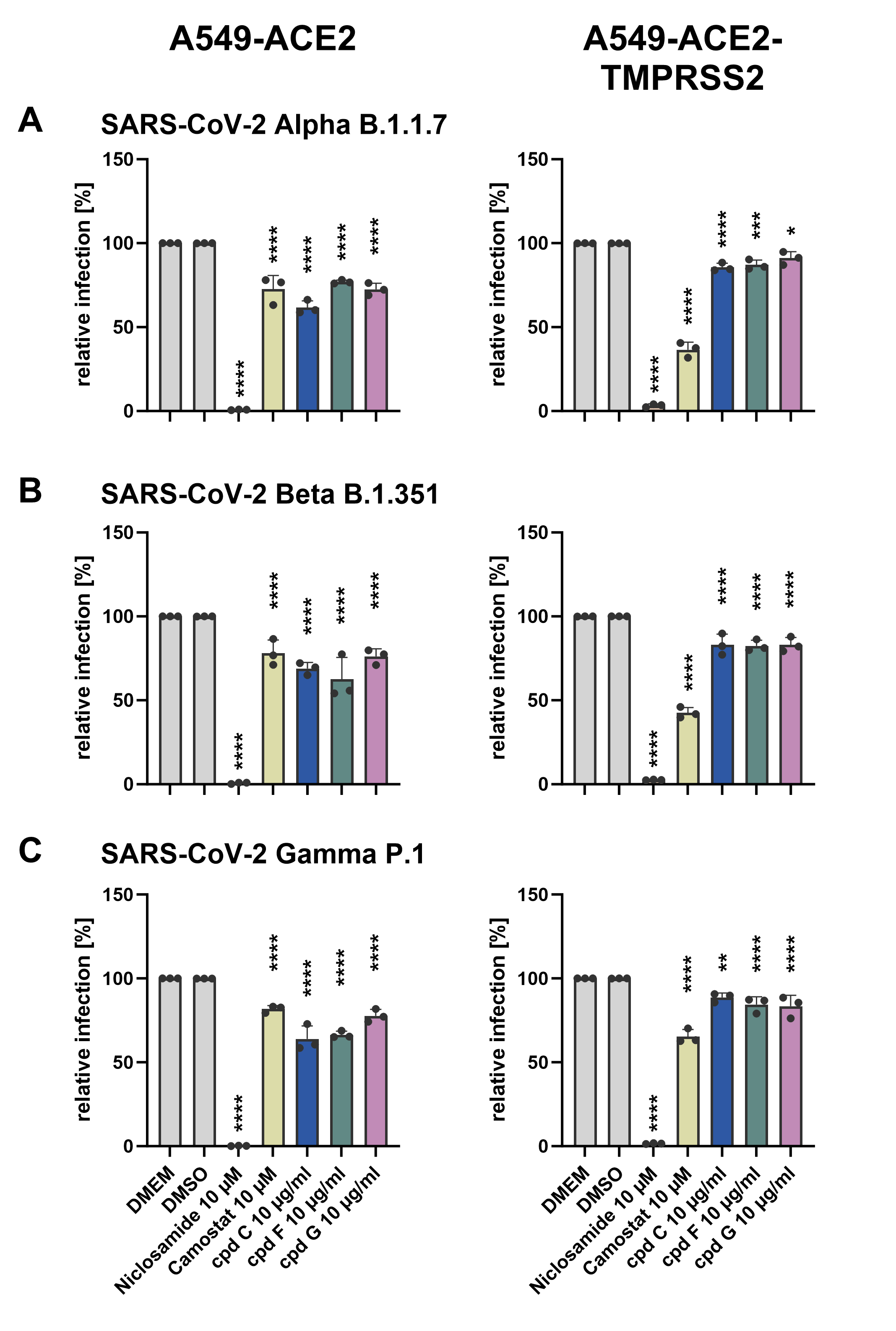

Supplement: Supplementary file 1 [file Image2.TIF]

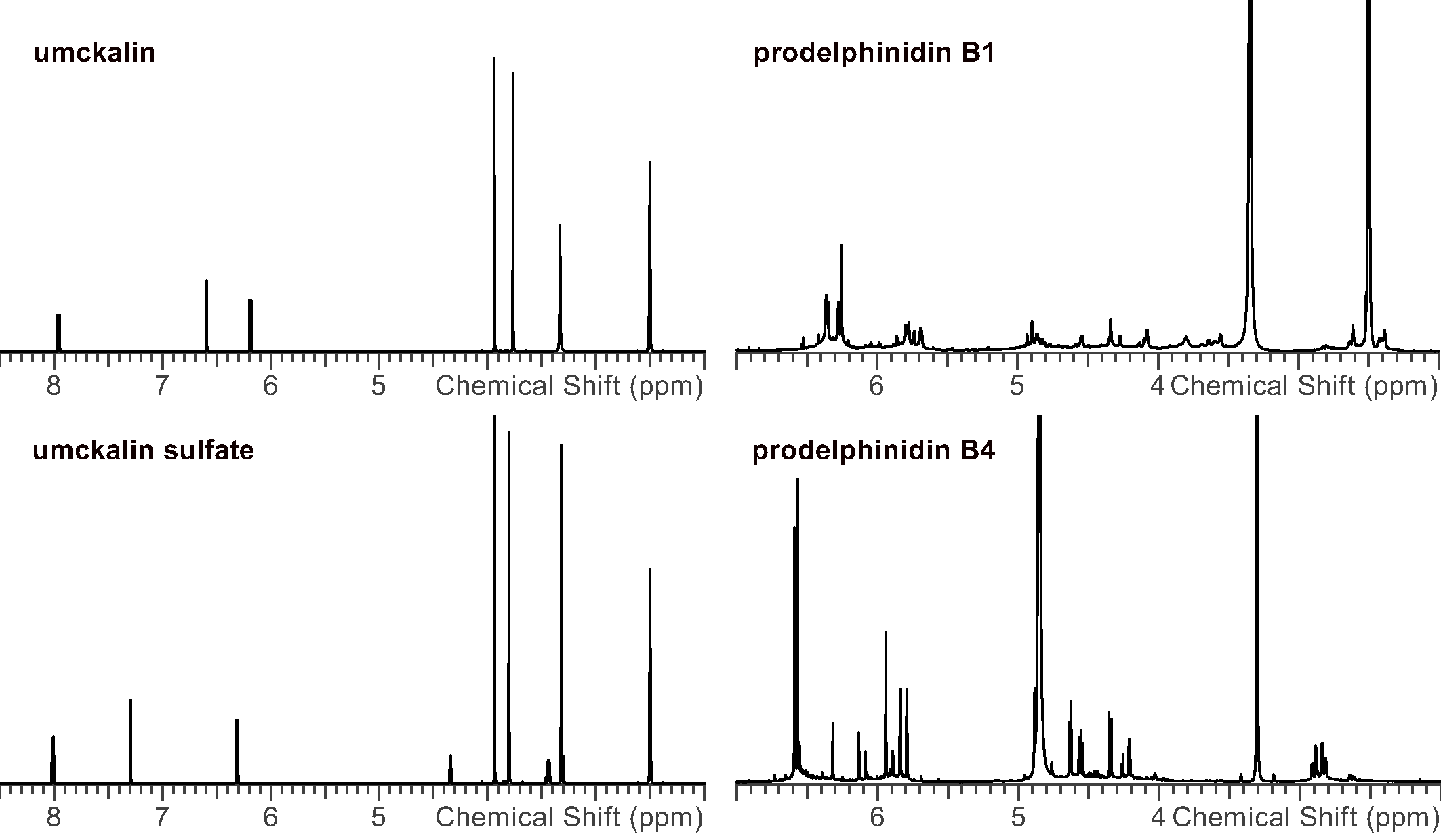

Supplement: Supplementary file 2 [file Image1.tif]
